# Fish oil improves hemodynamic stabilization and inflammation after resuscitation in a rat model of hemorrhagic shock

**DOI:** 10.1186/1476-511X-11-5

**Published:** 2012-01-10

**Authors:** Yang Li, Xinying Wang, Ning Li, Jieshou Li

**Affiliations:** 1 Research Institute of General Surgery, Jinling hospital, 305 Zhongshan East Road, Nanjing, 210002, China

**Keywords:** Hemorrhagic shock, Resuscitation, fish oil, Hemodynamic, Inflammatory response factor

## Abstract

**Background:**

Hemorrhagic shock followed by resuscitation stimulates an inflammatory response. This study tests the hypothesis that treatment with fish oil will attenuate inflammatory responses and stabilize hemodynamics.

**Methods:**

Male SD rats (n = 48; 250~300 g) were randomly divided into 4 groups: SHAM, hemorrhagic shock (HS), hemorrhagic shock/resuscitation (HS/R) and fish oil (FO). Shock was induced, and a mean arterial pressure (MAP) was maintained at 35 to 40 mmHg for 60 minutes. Resuscitation was carried out by returning half of the shed blood and Ringer's lactate solution to the animal. In FO group, fish oil (0.2 g/Kg) was infused through caudal vena at 30 minutes after shock. Half of each group was killed at 30 minutes and at 4 hours after resuscitation. Then several kinds of inflammation and oxidative stress indicators such as IL-6, MPO and GSH were tested.

**Result:**

FO group required less resuscitative fluid and had higher urinary output at the recovery periods from hemorrhagic shock than HS/R group(*p < 0.001*). After resuscitation, the MAP of HS/R group markedly declined than FO group (*p < 0.001*). The inflammatory indexes of FO group were lower than HS group and HS/R group and the same as sham group. But the level of endotoxin in FO group was significantly higher than sham group at 4 hours.

**Conclusion:**

Fish oil pretreatment before fluid resuscitation showed a beneficial effect to the hemodynamic stabilization and inflammation reduction in HS/R rat model.

## Introduction

Hemorrhagic shock (HS) remains a major cause of death in both civilian and combat traumatic injury[1]. It followed by resuscitation is considered as an insult frequently induces a systemic inflammatory response syndrome (SIRS) that results in multiple-organ dysfunction syndrome (MODS)[2], which is a major clinical problem. HS represents the acute hypovolemia and hemodynamic derangement and the mechanism is recognized to be the increasing of reactive oxygen species generation, and endothelial activation after supplementation with ambient oxygen in a prior ischemic field, and inducing many systemic inflammatory factor released[3-5].

Fish oil as pharmacological nutrients could directly and indirectly decreasing the production of inflammatory eicosanoids, cytokines, and reactive oxygen species and the expression of adhesion molecules[6]. Pscheidl EM et al[7] and Pscheidl E et al[8] have shown that fish oil not only attenuates inflammatory reactions, but ameliorates host defense and even improves splanchnic blood flow. Anti-inflammatory properties and benefit in splanchnic blood flow by fish oil could be protective for these tissue in ischemia/re-perfusion (I/R) [9,10]. Accordingly, this maybe a new approach to alleviate inflammation and hemodynamic stabilization in HS treatment. However, almost researches were focused on the pre-feeding of fish oil and few articles were after HS treatment.

So, our hypothesis was that infusion of fish oil before resuscitation in hemorrhagic shock rats, would help to minimize the post-resuscitation inflammatory responses and to stabilize hemodynamics.

## Animals and methods

### Rat model of Hemorrhagic Shock and Resuscitation

Forty-eight male SD rats of a mean(± SD) weight of 269.26 ± 12.68 g were used. Animals were provided by Medical Experiment Animal Center of Jinling Hospital(Nanjing, China). Temperature range between at 25°C-28°C with a 12:12-h light-dark cycle. Rats were allowed free access to water and chow. The protocol were approved by the institutional animal care committee.

HS/R model: All rats were anesthetized with 60 mg/kg ketamine intramuscular injection and placed on a temperature-controlled heating pads, which was heated while shock was induced and temperature was sustained 37°C. The left femoral artery and femoral vein were exposed and cannulated through a short incision[11]. Heparin (200 U/kg) was infused immediately through femoral vein following cannulation. The artery catheter was connected to a multi-channel recorder (Electronics for Medicine, TX) for the monitoring of systolic, diastolic, and mean arterial pressures (MAP) per 3 minutes, heart rate(HR) was also measured. The target MAP(35 ~ 40 mm Hg) was induced by removing blood slowly through the femoral vein cannula during a total time of 15 minutes and maintained at 35 to 40 mm Hg for 60 minutes. The animals in shock group were induced without resuscitation and killed at the end of shock instantly. At the point of 75 minute, resuscitation which would take 30 minutes and begin by returning half of the shed blood to the animal at the speed of 0.5 mL/min and giving Ringer's solution was carried out in HSR group and FO group. And Infusion was withheld if MAP exceeded 90% of baseline and re-started if declined. The rats in FO group were injected by fish oil (0.2 g/Kg) through the caudal vena at 45th minute. The rats in sham group were cannulated without inducing of shock and kept anesthetized for a same length of time with other groups'. At the end of 30 minutes of resuscitation, half of the animals of these three groups were randomly sacrificed to collect blood samples, and the others were killed at the end of 4 hours. The 2-3 ml sample was centrifuged at 4°C. After centrifugation, serum was kept refrigerated at -80°C until assayed. The volumes of fluid given and urine produced by the end of resuscitation period were recorded.

All animals were killed while anesthetized using aortic transaction.

### MDA MPO and GSH assays

To carry out the assays, the serum was assayed spectrophotometrically for malondialdehyde (MAD), myeloperoxidase(MPO) activity and reduced glutathione(GSH) level with assay kits (Nanjing Jiancheng Bioengineering Institute, Nanjing, China). All procedures were done in accordance with the manufacturer's instructions.

### Measurement of IL-6, TNF-α, Endotoxin by enzyme-linked immunosorbent assay

The blood sample was centrifuged at 10 000 xg for 15 min at 4°C. The supernatant was assayed for interleukin (IL)-6, Tumor necrosis factor α (TNF-α), Endotoxin levels by enzyme-linked immunosorbent assay kits (R&D Systems, Minneapolis, MN, USA) in accordance with the manufacturer's instructions. The absorbance at 450 nm was determined using a micro-plate reader (Tecan's, Sunrise, Unterberg strasse IA, Austria).

### Statistical analysis

MAP, concentrations of oxidative and pro-inflammatory mediators were expressed by their mean ± SD. Comparisons of various oxidative and inflammatory molecules, blood, and additional fluids volumes and MAP between the 4 groups were performed by ANOVA test. p < 0.05 was considered statistically significant.

## Results

### Volume of blood loss, resuscitation fluids and urine

The blood volumes withdrawn for the induction and maintenance of shock, resuscitation fluids, and urines are shown in Table 1. In this table we could find that during resuscitation the volume of Ringer's solution given in FO group was significantly less than in shock group(p < 0.001). But the volume of urines produced by FO group was dramatically higher when compared with shock and resuscitation group(p < 0.001).

**Table 1:** Volumes of Blood Withdrawn of the Induction and Maintenance of Shock, Fluids Given, and Urines During Resuscitation(Median ± SE)

	**Sham**	**HS**	**HS/R**	**FO Group**	***p***
	
Volume loss for induction to shock (mL/kg of body weight)	-	9.97 ± 0.22	10.19 ± 0.92	10.23 ± 0.45	0.742
Volume loss for maintenance of shock (mL/kg of body weight)	**-**	11.53 ± 0.46	11.57 ± 1.13	12.43 ± 0.84	0.187
Ringer's solution (mL)	**-**	**-**	4.80 ± 0.59	4.05 ± 0.89	0.001
Urines (mL)	0.93 ± 0.11	0.14 ± 0.22*	0.65 ± 0.19*	0.87 ± 0.21	0.001

independent samples t-test used in Ringer's solution; ANOVA p* < 0.001 vs. corresponding FO group

### Mean arterial pressure (MAP)

**Figure 1:**
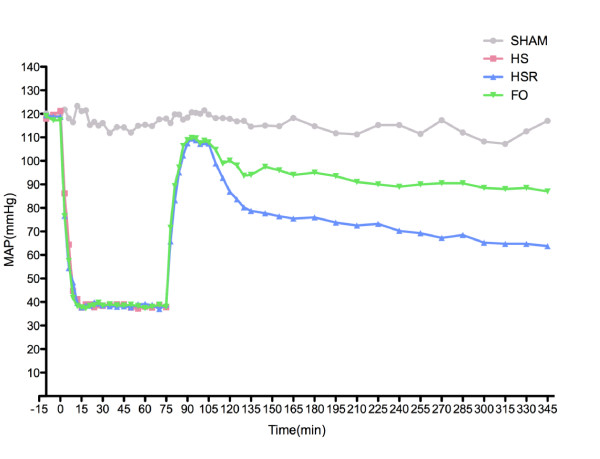
**Time of cost of mean arterial pressure(MAP)during shock and resuscitation**. Sham group remained at baseline; shock, HS/R, FO groups' MAP fell to 35-40 mmHg for 60 minutes. HS/R and FO groups recovered to baseline during resuscitation and declined gradually without intervention.

Figure 1 shows that MAP in the sham group did not change during the experiment. MAP in the shock group, resuscitation group, and FO group fell to the same level during shock. After pretreatment of fish oil the MAP was found no change in fish oil group. During resuscitation, the MAP maintained at 90% of baseline. After resuscitation, the decline was found in the two groups, but FO group had a smaller decrease than the HS/R groups did. After 105th min, MAP in FO groups had no dramatically change but had a significantly decline in HS/R group at each time points. (Table 2)

**Table 2:** After resuscitation, the MAP of sham group, resuscitation group and FO group in four different time points

	**4 Hours Post-resuscitation**			
	
	**Sham**	**HS/R**	**FO**	***p***
	
105 min	121.75 ± 8.54	109.00 ± 2.16	109.8 ± 2.17	0.36
155 min	122.25 ± 6.08*	76.5 ± 2.08*	96 ± 7.07	0.001
210 min	116.25 ± 4.86*	72.5 ± 2.88*	91.10 ± 1.41	0.001
345 min	123.00 ± 7.12*	63.75 ± 0.957*	87.05 ± 3.40	0.001
*p^a^*	0.504	0.001	0.11	

p* < 0.001 vs. corresponding FO group; *p^a ^: compared in the same group*

### Oxidative and Inflammatory Molecules

At 30 minutes post-resuscitation, the serum TNF-α was markedly elevated in HS, HS/R and FO groups but not in the sham group. The serum IL-6 and endotoxin was significantly increased in HS and HS/R groups when compared with sham group. Pretreatment with fish oil significantly decreased the serum IL-6 and Endotoxin levels in FO group when compared with the HS and HS/R groups.

At 4 hours post-resuscitation, TNF-α was raised in HS group and had significantly differences with sham and FO groups. HS and HS/R groups had a higher level of IL-6 than sham group did, and the level in HS/R group was markedly higher than FO group. Endotoxin in FO group was dramatically lower than HS/R group but significantly higher than sham and HS groups. The pro-inflammatory cytokines and endotoxin values in the different groups are shown in table 3.

**Table 3:** Mean Concentrations ± SE of the of Various Oxidative and Inflammatory Molecules

	**30 Minutes Post-resuscitation**				**4 Hours Post-resuscitation**			
	
	**Sham**	**HS**	**HS/R**	**FO**	**Sham**	**HS**	**HS/R**	**FO**
	
TNF-α (pg/mL)	59.53 ± 15.02	197.59 ± 18.33*	222.02 ± 13.21*	243.0 ± 10.2*	42.00 ± 1.08	197.59 ± 18.33* #	66.07 ± 9.03	43.31 ± 0.084
IL-6 (pg/mL)	21.71 ± 0.78	84.80 ± 5.66* #	122.01 ± 24.72* #	22.26 ± 2.35	22.80 ± 1.06	84.80 ± 5.66*	122.85 ± 31.23* #	54.89 ± 5.22
Endotoxin(EU/mL)	0.22 ± 0.009	0.87 ± 0.056* #	1.26 ± 0.116* #	0.24 ± 0.016	0.29 ± 0.046	0.87 ± 0.056* #	3.60 ± 0.358* #	2.58 ± 0.24*
Total GSH (μmol/L)	703.75 ± 9.64	741.10 ± 31.76	714.96 ± 23.40	719.47 ± 13.0	680.77 ± 31.9	741.1 ± 31.77	719.47 ± 16.65	717.1 ± 16.89
Reduced-GSH (μmol/L)	273.71 ± 15.4	259.00 ± 5.83	255.91 ± 3.87	262.84 ± 21.2	283.31 ± 8.02	259.00 ± 5.83	254.33 ± 8.14	269.42 ± 3.48
Reduced/total GSH	0.78 ± 0.047	0.70 ± 0.033	0.72 ± 0.013	0.73 ± 0.06	0.81 ± 0.073	0.70 ± 0.033	0.70 ± 0.033	0.75 ± 0.060
MDA (μmol/L)	15.72 ± 11.59	43.91 ± 1.96* #	52.55 ± 5.22* #	27.78 ± 4.54*	12.32 ± 5.16	43.91 ± 1.96* #	59.67 ± 12.87* #	17.55 ± 5.14
MPO(μmol/L)	26.55 ± 3.61	199.11 ± 18.06* #	257.74 ± 12.18* #	172.5 ± 58.3*	27.50 ± 8.08	199.11 ± 18.06* #	356.93 ± 9.21* #	61.95 ± 6.26

* P < 0.01 vs sham group (1-way ANOVA); *^# ^P < 0.01 *shock and resuscitation group vs FO group

The levels of total GSH, reduced GSH and reduced/total GSH had no difference at the two time points in the four groups. The MDA levels, as a major degradation product of lipid per-oxidation, were found to be significantly higher in HS, HS/R and FO groups as compared with those of the sham group at 30 min post-resuscitation, while treatment with FO abolished these elevations at 4 hours. MPO, a marker of neutrophils, had the same tendency as MDA.

## Discussion

Hemorrhagic shock is considered as an insult frequently leading to systemic inflammatory response syndrome including the systemic release of pro-inflammatory cytokines. And an increase in systemic pro-inflammatory cytokines was associated with cellular injury and remote organ dysfunction[12-14]. On this concept, the routine clinical medical treatment should minimize excessive inflammatory reaction induced by ischemia/re-perfusion. Many animal studies published so far, have shown the advantage of fish oil on ischemia/re-perfusion and hemorrhagic shock/resuscitation through alleviating the inflammatory response and organ protection. Similarly, several clinical studies have confirmed that fish oil may decrease inflammatory cytokine release in several diseases[15] and improves splanchnic blood flow[7,8]. However, the benefits of clinical use of fish oil in HS/R patients remains unclear, which in part prompted the present study.

The present study found that during shock induction the blood volume withdrawn were same in every group. And the volume in FO group at shock maintenance was slightly more than the other groups', although without statistically significant(Table 1). The probable reason is that the infusion of fish oil increased volume loss for maintenance of shock in FO group.

The use of fish oil was associated with hemodynamic stability. In fact, the FO group required less resuscitation fluid than HS/R group, and had higher urinary output at the recovery from hemorrhagic shock than HS and HS/R groups (Table 1). In Figure 1, we could find that there was no difference in MAP among the groups at the end of resuscitation, but after that the MAP of HS/R group and FO group both dropped without intervention. So the four time points were selected to analyze the MAP in each group (Table 2). Except the 105th min, at other three time points, the MAPs in FO group were higher than HS/R group's. Meanwhile, the MAP of HS/R group was dramatically decreased and significantly less than the MAP in the previous time points. It may be argued that it merely was the extra infusion of fish oil improved the MAP in FO group. There are three reasons advocating that not the infusion of itself, but the pharmacological effects of fish oil worked. Firstly, during the infusion of fish oil, the MAP was maintained by withdrawing and infusion blood through femoral vein and the loss volumes during shock have no significantly difference between HS/R group and FO group. Secondly, at the end of resuscitation each group has the same MAP. Thirdly, the intravenous supply of fish oil can lead to a change in membrane FA patterns after a short time(3-5 hours)[16,17], exerts anti-inflammatory and alters spectrum of metabolites in the situation of inflammatory stimulation. Moreover, the bolus administration of FO solution in rats achieves incorporation of n-3 PUFAs into cell membranes after only 60 min [18]. So, these three causes provide a credence to the aspect that the pharmacological effects rather than the infusion itself ameliorated the MAP.

Additionally, at 30 minutes post-resuscitation, the levels of IL-6, MDA and MPO in FO group were significantly lower than HS group's and HS/R group's, but this difference was not found in TNF-α. At 4 hours post-resuscitation, the levels of TNF-α, MDA and MPO in FO group were dramatically lower than those in HS group, and the levels of IL-6, MDA and MPO were lower than HS/R group's. The "classic" explanation for the effects of fish oil on regulation of inflammatory host responses is that the administration of fish oil inhibits the production of pro-inflammatory cytokines, such as TNF-α, IL-1β and IL-6, and seems to positively modulate the production of the anti-inflammatory cytokine IL-10[19,20]. Ertel et al[21] pre-fed mice with fish oil for 3 weeks, subjected them to HS/R, and harvested peritoneal macrophages at 24 hours post-resuscitation. They found that fish oil prevented an increase in levels of circulating prostaglandin E2 (PGE2) release seen in mice fed with corn oil or safflower oil.

In this study, we observed that the concentration of endotoxin in FO group was significantly lower than shock group and HS/R groups. The reason should be that intestinal ischemia/reperfusion results in damage to the intestinal mucosa, with loss of barrier function for endogenous bacteria. This means that bacteria and toxins from the intestine can be translocated into the bloodstream and other organs[22]. But the administration of fish oil promoted intestinal blood flow in the rats and thus possibly prevented the destruction of intestinal barrier function associated with ischemia/reperfusion[7]. A study with endotoxemia rats confirmed the positive effects of fish oil infusion on the microcirculation in the splanchnic area, and the number of viable bacteria in the liver and small bowel lymph node were significantly reduced[8].

At the same time, we found that the concentration of endotoxin in FO group at 4 hours point was dramatically higher than sham group, however there is no difference in oxidative stress and Inflammatory indexes between them. Why the rats in FO group have slightly pathological changing with obvious endotoxemia? This phenomenon might ascribe to the blunting of the response to endotoxin and reducing of inflammatory release. Pittet. YK et al[23] have proved that 0.2 g/Kg perfusion of fish oil immediately (2 hours before endotoxemia) can blunted the response to endotoxin in healthy subjects after 3 hours. Once fish oil administrated, the fatty acids it contained would incorporate into cell membrane phospholipids and play key roles in physicochemical and functional characteristics of the cell membranes. It can modify macrophage membranes and reduce its phagocytosis and adhesion function, and decreases the mortality and severity of SIRS[24]. Mayer et al.[25] have shown that several hours of parenteral nutrition with fish oil significantly decreases the ω-6/ω-3 FA ratio in the plasma-free FA fraction and in the monocyte membrane lipid pool which was associated with a suppression of monocyte generation of pro-inflammatory cytokines (TNF-α, Interleukin-1, -6, -8) in response to endotoxin. Oz et al[26] showed that 6 days of a fish oil diet could protect rats from the hepatic damage associated with lipopolysaccharide (LPS) administration.

In this study, the HS rats were resuscitated with half of the shed blood and Ringer's solution and administered fish oil before resuscitation in order to ensure the model simulates clinical practicing. The results from the present study support our hypothesis that treatment with fish oil can alter the inflammatory response to HS/R, blunt the response to endotoxin and improve the hemodynamic stabilization after resuscitation.

## Conclusions

Our study firstly demonstrates that infusion of fish oil before resuscitation not only can alter the inflammatory response to HS/R but also blunt the response to endotoxin. Finally, the alleviation of inflammatory response and endotoxemia results in hemodynamic stabilization.
